# Tim-3 Expression Causes NK Cell Dysfunction in Type 2 Diabetes Patients

**DOI:** 10.3389/fimmu.2022.852436

**Published:** 2022-04-05

**Authors:** Hui Wang, Kangli Cao, Siyu Liu, Yuanhong Xu, Ling Tang

**Affiliations:** Department of Clinical Laboratory, The First Affiliated Hospital of Anhui Medical University, Hefei, China

**Keywords:** type 2 diabetes mellitus, NK cells, Tim-3, dysfunction, apoptosis

## Abstract

Type 2 diabetes mellitus (T2DM) is characterized by high blood glucose levels and chronic low-grade inflammation. It shows a strong association with obesity and immune dysfunction, which makes T2DM patients more susceptible to infectious diseases. NK cells play an important role in pathogen control and tumor surveillance. However, whether NK cell distribution and functional status are altered in T2DM is unclear. To address this issue, we compared surface receptor expression and cytokine production between peripheral blood NK cells from 90 T2DM patients and 62 age- and sex-matched healthy controls. We found a significantly lower frequency and absolute number of NK cells in patients than in controls. Interestingly, the expression of inhibitory receptor Tim-3 was significantly increased, while the expression of the activating receptor NKG2D was significantly decreased, in T2DM NK cells. Both TNF-α secretion and degranulation capacity (evidenced by CD107a expression) were dampened in NK cells from patients. The expression of Tim-3 on NK cells correlated positively with both HbA1c and fasting blood glucose levels and negatively with the percentage and absolute number of total NK cells and was associated with increased NK cell apoptosis. In addition, Tim-3 expression on NK cells negatively correlated with TNF-α production, which could be restored by blocking Galectin-9/Tim-3 pathway. Our results suggest that NK cell dysfunction secondary to augmented Tim-3 expression occurs in T2DM patients, which may partly explain their increased susceptibility to cancer and infectious disease.

## Introduction

There are approximately 537 million diabetic patients across the world in 2021, of which more than 90% suffer from type 2 diabetes mellitus (T2DM) (1). T2DM is a chronic disease characterized by hyperglycemia, insulin resistance, and low-grade inflammation (2, 3). T2DM is thought to cause immune dysfunction, compromising the ability of patients to control the spread of invading pathogens. As a result, people with T2DM are more vulnerable to infectious disease, leading to increased morbidity and mortality (4, 5).

Natural killer (NK) cells play an important role in immune surveillance against viruses and cancers (6). NK cells express a variety of inhibitory receptors, including T-cell immunoglobulin and mucin domain-containing protein 3 (Tim-3), TIGIT, NKG2A, and LAG-3, as well as activating receptors, including NKG2D, NKp30, and NKp46 (7). NK cells recognize infected or transformed cells *via* their inhibitory receptors through a “missing-self” mode, i.e., by detecting the absence on the surface of target cells (8). Human NK cells are CD3^-^/CD56^+^ large granular lymphocytes and can be divided into CD56^dim^ and CD56^bright^ subtypes based on surface CD56 expression (9). CD56^dim^ NK cells account for approximately 90% of total circulating NK cells and mediate tumor lysis through the secretion of perforin and granzyme (10). The proportion of CD56^bright^ NK cells in the peripheral blood increases notably at inflammation sites, where they become activated and produce a variety of cytokines, notably IFN-γ and TNF-α (10).

Previous reports on the influence of T2DM on NK cells are limited and conflicting. Barrou et al. reported a significant reduction in the frequency of both NKp46^+^ and NKG2D^+^ NK cells in T2DM (11). Piątkiewicz et al. showed that NK cell counts are increased, but their cytotoxic function is impaired in T2DM (12). In contrast, Lim et al. reported that neither abundance nor activity of NK cells is altered in patients with long-term T2DM (13).

Tim-3 is an immune inhibitory receptor expressed on NK cells, T cells, and mast cells (14). Upon binding to its ligands, Galectin-9 and CD66a (also known as CEACAM1), Tim-3 exerts an immune inhibitory function (15, 16). Studies have shown that increased Tim-3 expression on NK cells during chronic viral infections and cancer is associated with NK cell exhaustion (17–20). Therefore, Tim-3 is a potential therapeutic target for oncological disease, and several drugs targeting this receptor are being tested in preclinical and clinical studies (21, 22).

The expression of Tim-3 is upregulated in both CD4^+^ T cells and CD8^+^ T cells from T2DM patients (23). In turn, increased serum Galectin-9 levels were reported in T2DM (24). While these results suggest that T2DM leads to T cell dysfunction, it remains unclear whether T2DM also affects the function of NK cells. Therefore, to further explore potential changes in NK cell distribution and function in patients with T2DM, we analyzed the expression of inhibitory and activating receptors, as well as cytokine production, in circulating NK cells isolated from T2DM patients and healthy controls. Our results indicate that T2DM is associated with NK cell dysfunction and apoptosis, mediated by increased expression of Tim-3. These findings may explain the increased susceptibility to infectious disease and cancer of T2DM patients and suggest that Tim-3 is a potential target for T2DM therapy.

## Materials and Methods

### Patients and Healthy Controls

The study protocol was approved by the Ethics Committee of the First Affiliated Hospital of Anhui Medical University and written informed consent was obtained from each individual. This study included 90 type 2 diabetes patients and 62 age- and sex-matched healthy controls (HCs). Clinical characteristics of the patients and controls are shown in **Supplementary Table S1**. The inclusion criterion was more than 5 years of diabetes. Exclusion criteria included stage 3 and 4 chronic kidney disease, history of malignancy, acute diseases, immunosuppressive therapy, and chronic or acute hepatitis B or AIDS. In addition, female and male subjects aged under 40 or over 80 years were excluded. The diagnostic criteria for type 2 diabetes are based on the World Health Organization (WHO) diagnostic criteria (http://www.who.int/diabetes/publications/diagnosis_diabetes2006/en/).

### Isolation of PBMCs and Flow Cytometry

**Figure 1:**
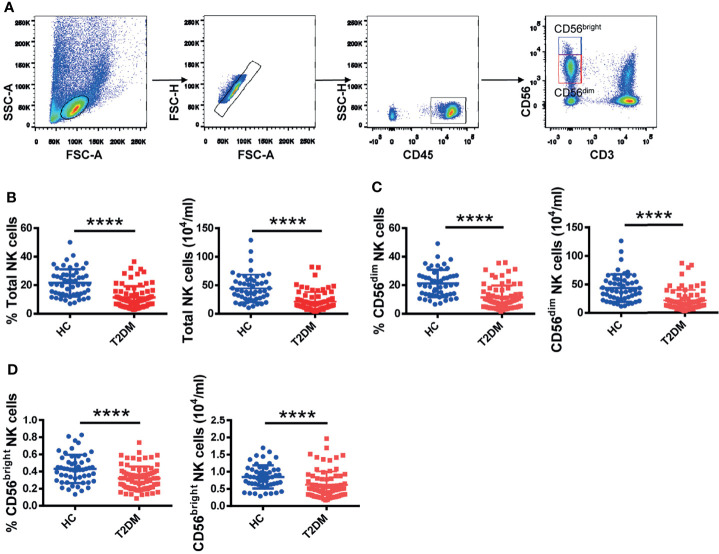
Circulating NK cell counts are decreased in T2DM patients. **(A)** Sequential strategy for gating CD56^dim^ and CD56^bright^ NK cells from PBMCs using flow cytometry. Flow cytometry estimations of frequency and absolute number of total NK cells **(B)**, CD56^dim^ NK cells **(C)**, and CD56^bright^ NK cells **(D)** in patients with T2DM and healthy controls (HC). Each dot represents a different individual and results are presented as the mean ± SEM; ****p < 0.0001.

Peripheral blood mononuclear cells (PBMCs) were separated by density gradient centrifugation using human peripheral blood lymphocyte isolation fluid. (TBD Science, Tianjin, China). For flow cytometry staining of PBMCs, the following anti-human monoclonal antibodies were obtained from BioLegend:CD3 (HIT3a, PerCP-Cy5.5), CD3 (HIT3a, FITC), CD56 (HCD56, BV510), CD56 (HCD56, PE-Cy7), CD45 (2D1, APC-Cy7), Tim-3 (F38-2E2, PE), NKG2A (S19004C, APC), TIGIT (A15153G, APC), NKG2D (1D11, APC), CD16 (3G8, APC), NKP46 (9E2, FITC), CD27 (M-T271, FITC), CD69 (FN50, PE-Cy7), CD14 (63D3, PE), Galectin-9 (9M1-3, PE-Cy7), CD4 (OKT4, APC-Cy7), LAG-3 (11C3C65, BV421), TNF-α (W19063E, PE), IFN-γ (4S.B3, APC), and CD107a (H4A3, PE-Cy7). Isotype control immunoglobulin G was used as control. Anti-CD66 (B1.1, FITC), anti-CD19 (HIB19, PerCP-Cy5.5), anti-Ki67 (B56, Alexa-Fluor 647) and anti-HLA-DR (L203.rMAb, PE) was purchased from BD Bioscience. PBMCs were incubated with antibodies at 4°C for 30 min. Cell staining was evaluated using a BD FACSCanto flow cytometer, and data analysis was performed with FlowJo software v10 (TreeStar, USA). For intracellular cytokine assays, isolated PBMCs were stimulated with medium containing 10% fetal bovine serum (FBS, Biosharp, China) in the presence of 50 ng/ml phorbol 12-myristate 13-acetate, 1 µg/ml ionomycin, and 10 µg/ml monensin (all from Sigma-Aldrich, USA) for 4 h. Following immunolabeling of surface molecules, cells were fixed, permeabilized, and labeled with specific cytokine-targeting antibodies. After washing off excess antibodies, signal detection was carried out by flow cytometry. The gating strategy is shown in **Figure 1**.

### Lentiviral Package and Transduction of Cells

pLVX-EF1α-IRES-puro vector containing human Galectin-9 and CD66a gene was generated by Azenta. Lentiviral supernatants were generated by co-transfecting HEK293T cells with pLVX-EF1α-IRES-puro, psPAX2 (VT1444, YouBio, Changsha, Hunan, China) and pMD2G (VT1443, YouBio, Changsha, Hunan, China) plasmids using Lipofectamine 3000 reagent (Invitrogen, Carlsbad, CA, USA). Culture supernatants collected 48 h after transfection were added directly to K562 cells plated in 6-well culture plates, and Polybrene (Sigma Aldrich, St. Louis, MO, USA) was added at a final concentration of 5 μ g/mL for 24 h. Transduced cells were selected by adding 5 μ g/mL puromycin (Invitrogen, Carlsbad, CA, USA). K562 cells transduced with Galectin-9 and CD66a were named K562-Galectin-9 and K562-CD66a, respectively. Transfection efficiency was determined by flow cytometry.

### *In Vitro* Culture Systems

#### Tim-3 and Galectin-9 Blockade

For Tim-3 blocking assays, PBMCs from T2DM patients were preincubated with 10 μg/ml of anti-human Tim-3 (BioLegend, Cat#F38-2E2) or Galectin-9 (BioLegend, Cat#9M1-3) in DMEM (Hyclone, USA) containing 10% T2DM serum for 6 h at 37°C. These cells were subsequently stimulated with 50ng/ml IL-12 plus 50ng/ml IL-18 (Proteintech, USA) for evaluating cytokine secretion and granule release.

#### PBMC Culture Systems

Freshly isolated PBMCs from healthy donors were cultured in glucose-free RPMI-1640 medium (Procell, China) with 10% FBS containing 5.5 mM, 25 mM and 50 mM glucose (25). After 24h, 48h and 72h, the expression of Tim-3 on NK cells was analyzed by flow cytometry.

#### NK Cell-Mediated Cytotoxicity Assay

NK92 cells or freshly isolated PBMCs from T2DM patients were diluted in RPMI-1640 medium (Procell, China) containing 10% FBS and seeded into 96-well round bottom plate. K562, K562-Galectin-9, K562-CEACAM-1 cells were labeled with CellTrace Violet Cell Proliferation Kit (Thermofisher, C34557) according to manual protocol, diluted in RPMI-1640 medium (Procell, China) containing 10% FBS and then seeded into 96 well round bottom plate (1×10^4^/well) with or without effector cells. Tim-3 antibody or Galectin-9 antibody was added to a final concentration of 5μg/ml at the final volume of 200μl/well. The plate was then cultured at 37°C, 5% CO_2_ for 4h. After 4 h, the wells were harvested and 7-aminoactinomycin D (7-AAD, BD bioscience) was added before analyses. Samples were mixed thoroughly and analyzed by flow cytometry.

#### NK92 and Target Cell Coculture

1×10^4^ NK92 were cultured in RPMI-1640 medium (Procell, China) containing 10% FBS with K562, K562-Galectin-9 and K562-CEACAM-1 cells at the E:T ratio of 1:1 in 96 well round bottom plate at the final volume of 200μl/well. After 24h, the culturing supernatants were collected and analyzed by human IFN-γ ELISA kit (Dakewe Biotech, 1110002) according to manual protocol.

### Apoptosis Detection

Apoptosis detection in NK cells was performed using an APC Annexin V Apoptosis Detection Kit (BioLegend). Cells were washed twice with PBS and resuspended in 1× binding buffer at a concentration of 1×10^6^ cells/ml. Then, a 100 µL sample (1×10^5^ cells) was transferred to a 5 ml test tube with 5 µL APC Annexin V and 7-AAD. The cells were then gently vortexed and incubated for 15 min in the dark at room temperature (25°C). Finally, 400 µL of 1× binding buffer was added to each tube before analysis by flow cytometry.

### ELISA

The level of Galectin-9 in the human serum was measured using Human Galectin-9 ELISA kit (Proteintech, USA) according to the manufacturer’s protocol.

### Statistical Analysis

Statistical analysis was performed using GraphPad Prism 9.0 software. Quantitative data were expressed as mean ± standard deviation (SD). Comparisons were made between two groups using an unpaired t-test or the Mann-Whitney U test, depending on whether group values conformed to a normal distribution. Pearson’s correlation test was used to identify correlations between cell receptor expression and biochemical variables. P<0.05 was considered significant.

## Results

### T2DM Patients Exhibit a Reduced Number of Peripheral Blood NK Cells

To investigate whether NK cells are affected in T2DM patients, we recruited 90 patients with T2DM and 62 age- and sex-matched healthy volunteers and analyzed their PBMCs. Gating strategies for total lymphocytes and NK cells are shown in **Supplementary Figure S1A**. We found that the frequency and absolute number of total NK cells were significantly lower in patients with T2DM compared to healthy volunteers (**Figure 1B**). We further analyzed the distribution of the two NK cell subpopulations, i.e., CD56^dim^ and CD56^bright^ NK cells (see **Figure 1A** for details). In line with the observed changes in total NK cell numbers, the frequency and absolute number of CD56^dim^ and CD56^bright^ NK cells were also significantly lower in T2DM patients (**Figures 1C, D**). No significant differences between groups were noted in the analysis of CD3^+^ T lymphocytes and CD14^+^ monocytes (**Supplementary Figure S1B**).

### NK Cells From T2DM Patients Overexpress Tim-3 and Underexpress NKG2D

**Figure 2:**
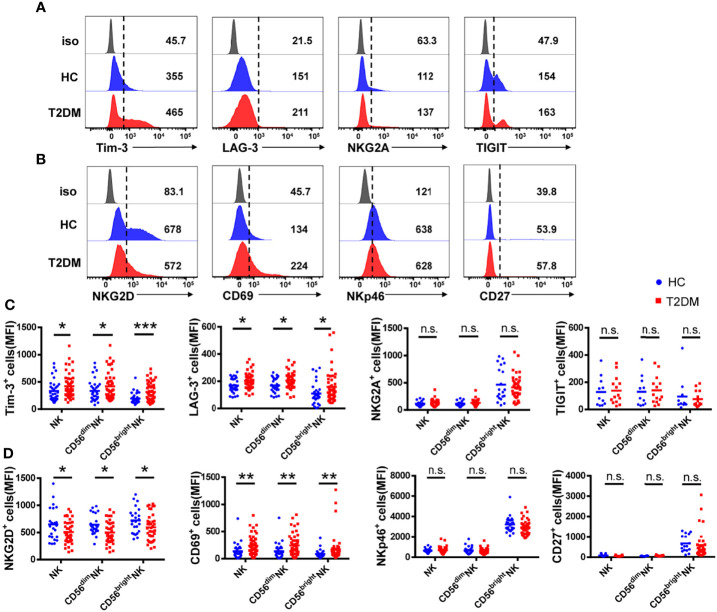
NK cells from T2DM patients have an impaired phenotype. **(A)** Representative histograms showing the expression of the inhibitory receptors Tim-3, LAG-3, NKG2A, and TIGIT on total NK cells from patients with T2DM and healthy controls (HC). **(B)** Representative histograms showing the expression of activating receptors NKG2D, CD69, NKp46, and CD27 on total NK cells from patients with T2DM and HC. **(C)** Comparison of the mean fluorescence intensity (MFI) of inhibitory receptor expression in total NK cells and NK cell subsets. **(D)** Comparison of the MFI of activating receptor expression in whole NK cells and NK cell subsets. Data are representative of more than three independent experiments and results are presented as the mean ± SEM; *p < 0.05, **p < 0.01, ***p < 0.001, n.s., not significant. See also **Supplementary Figure S2**.

To determine whether T2DM affects receptor expression on NK cells, we analyzed the expression of the inhibitory receptors Tim-3, NKG2A, LAG-3, and TIGIT, as well as the expression of the activating receptors NKG2D, NKp46, and CD69 on peripheral blood NK cells. We found that the mean fluorescence intensity (MFI) and the frequency of Tim-3^+^ and LAG-3^+^ NK cells were increased, while the expression of NKG2A and TIGIT was not affected (**Figures 2A, C** and **Supplementary Figure S2A**). In line with data from total NK cells, overexpression of both Tim-3 and LAG-3 was detected in both CD56^dim^ and CD56^bright^ NK cell subsets. MFI and frequency of NKG2D^+^ NK cells were decreased in T2DM patients, while activating receptor NKp46 expression was not affected (**Figures 2B, D**, and **Supplementary Figure S2B**). Similar expression patterns for these two markers were also recorded in CD56^dim^ and CD56^bright^ NK cell subsets. Indicating NK cell activation, the expression of CD69 was upregulated in samples from T2DM patients compared to controls (**Figure 2D**). We also analyzed the expression of CD27, a marker of NK cell differentiation and maturation (26). Results showed that CD27 expression was significantly higher in CD56^bright^ compared to CD56^dim^ NK cells in both diabetic and non-diabetic subjects (**Figure 2D** and **Supplementary Figure S2B**).

Among the inhibitory receptors assessed, Tim-3 was the most abundantly expressed on NK cells (**Figure 2C**). In turn, both MFI and percentage of Tim-3^+^ NK cells were higher in T2DM patients relative to controls. Consistent with previous studies (24), there was no significant difference in Tim-3 expression on CD3^+^ T cells from T2DM patients compared to healthy volunteers (**Supplementary Figure S2C**). These results suggest that peripheral blood NK cells in T2DM patients are under chronic stimulation and proceed into an immunosuppressed state, denoted by increased Tim-3 and decreased NKG2D expression.

### T2DM Patients Have Impaired NK Cell Functions

**Figure 3:**
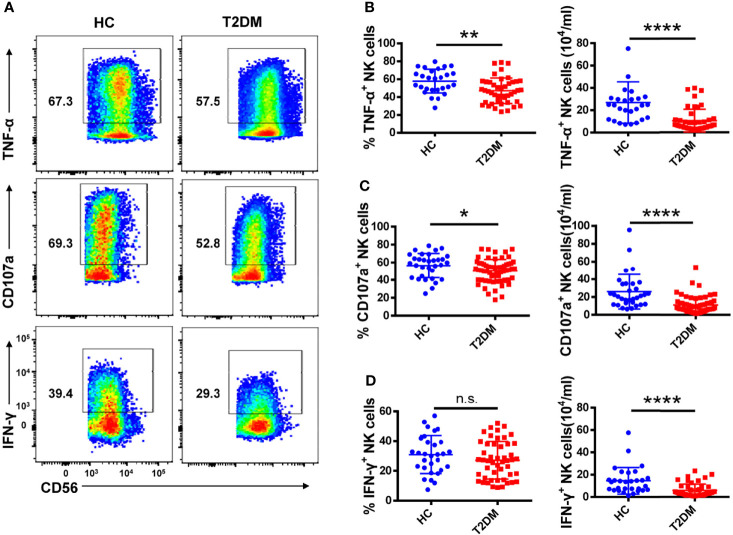
Circulating NK cells from patients with T2DM show functional impairment. **(A)** PBMCs from T2DM and healthy controls (HC) were stimulated with PMA and ionomycin and the expression of TNF-α, IFN-γ, and CD107a by NK cells was determined by flow cytometry. **(B–D)** Analysis of the percentage and absolute number of TNF-α^+^ **(B)**, CD107a^+^ **(C)**, and IFN-γ ^+^ **(D)** NK cells in patients with T2DM and HC. Each dot represents a different individual and results are presented as the mean ± SEM; *p < 0.05, **p < 0.01, ****p < 0.0001, n.s., not significant.

To assess potential functional changes in NK cells in T2DM, we examined by flow cytometry their cytokine production capacity upon PMA/ionomycin stimulation (see **Supplementary Figure S3A** for gating details). As shown in **Figure 3A**, both the percentage and absolute number of TNF-α^+^ NK cells were significantly decreased in T2DM patients (**Figure 3B**). Moreover, NK cells from T2DM patients also exhibited impaired degranulation, as evidenced by decreased CD107a expression compared to healthy volunteers (**Figure 3C**). The percentage of IFN-γ^+^ NK cells was also decreased in T2DM patients, though this decrease did not achieve statistical significance, and the lower absolute number of IFN-γ^+^ NK cells may be attributed to their lower NK cells (**Figure 3D**). These data suggest that NK cells have reduced cytokine synthesis and are less likely to mediate cytotoxicity in patients with T2DM compared to healthy individuals.

### Increased Tim-3 Expression Correlates With Enhanced Apoptosis of NK Cells in T2DM Patients

**Figure 4:**
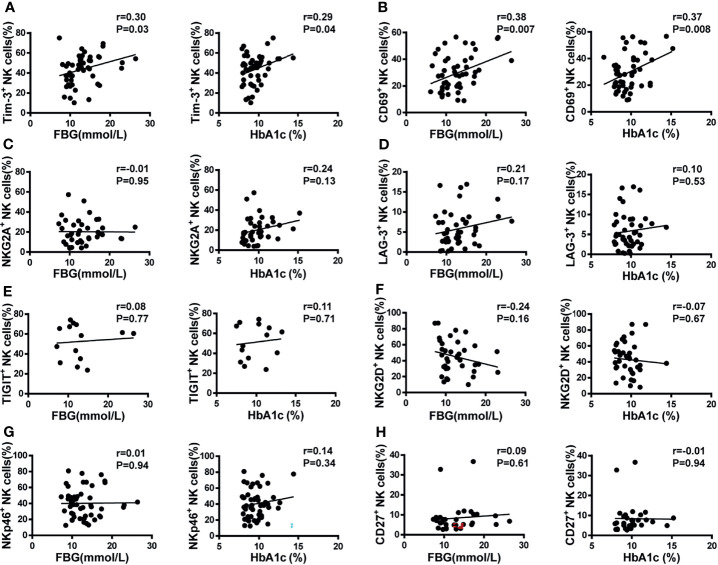
Pearson’s correlation analysis of NK cell receptor expression, fasting blood glucose (FBG), and glycosylated hemoglobin (HbA1c) in T2DM patients. **(A)** Scatter plot graphs indicating positive correlation between Tim-3^+^ expression and both FBG and HbA1c. **(B)** CD69^+^ NK cells were positively correlated with both FBG and HbA1c. No correlation was detected between the percentage of NKG2A^+^ **(C)**, LAG-3^+^ **(D)**, TIGIT^+^ **(E)**, NKG2D^+^ **(F)**, NKp46^+^ **(G)**, and CD27^+^ **(H)** NK cells and either FBG or HbA1c.

Glycosylated hemoglobin (HbA1c) is a standard indicator for glycemic control and prediction of diabetes complications (27). Patients with poorly controlled T2DM have higher HbA1c and elevated fasting blood glucose (FBG) levels. Therefore, the correlation between NK cell receptor expression and HbA1c and FBG levels was analyzed in the two cohorts. As shown in **Figure 4A**, Tim-3 expression on NK cells from T2DM patients was positively correlated with both HbA1c and FBG levels (r=0.29, P=0.04, and r=0.30, P=0.03, respectively). Likewise, and suggestive of NK cell activation, a positive correlation between CD69 expression and both HbA1c and FBG levels were also detected in T2DM samples (r=0.37, P=0.008 and r=0.38, P=0.007, respectively; **Figure 4B**). In contrast, no correlation between the expression of other NK cell receptors (NKG2A, LAG-3, TIGIT, NKG2D, NKp46, and CD27) and either HbA1c or FBG levels was observed in T2DM patient samples (**Figures 4C–H**). To further determine whether high concentrations of glucose affect the expression of Tim-3 on NK cells *in vitro*, PBMCs isolated from healthy donors were cultured in medium containing 5.5 mM, 25 mM, and 50 mM of glucose and Tim-3 expression was analyzed by flow cytometry. We found that high glucose treatment did not change the expression of Tim-3 directly (**Supplementary Figures S4A, B**).

**Figure 5:**
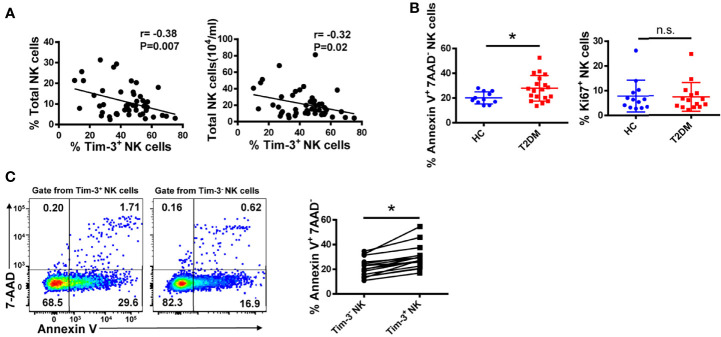
Tim-3 expression is associated with apoptosis of peripheral blood NK cells in T2DM. **(A)** Correlation analysis of Tim-3^+^ NK cells and percentage and absolute number of total NK cells in PBMC samples from patients with T2DM (Spearman’s correlation test). **(B)** Comparison of the early apoptosis (Annexin V^+^7-AAD^-^) and proliferation (Ki67^+^) of NK cells from T2DM patients and healthy controls (HC). Flow cytometry staining for Annexin V **(C)** of Tim-3^+^ versus Tim-3^-^ NK cells in T2DM PBMC samples. Quantification data for each test sample are shown in the right panel. *P < 0.05. n.s., not significant.

Despite its characteristic abundance, the regulatory function of Tim-3 on NK cells in the microenvironment of T2DM is unclear. Interestingly, we found that in NK cells from T2DM patients the expression of Tim-3 was negatively correlated with both the percentage and absolute number of circulating NK cells (r=–0.38, P=0.007 and r=–0.32, P=0.02, respectively; **Figure 5A**). Consistent with these data, we observed that the frequencies of Annexin V^+^ NK cells were higher in T2DM patients than in healthy donors, while the frequencies of proliferative (Ki67^+^) cells among two cohorts were similar (**Figure 5B**). Further analysis showed that Tim-3^+^ NK cells from T2DM patients showed a greater propensity to undergo apoptosis compared with Tim-3^-^ NK cells (**Figure 5C**). Overall, these results indicate that in poorly controlled T2DM, the expression of Tim-3 on NK cells is higher, which may cause NK cell dysfunction and apoptosis.

### Tim-3^+^ NK Cells Are Exhausted in T2DM Patients

**Figure 6:**
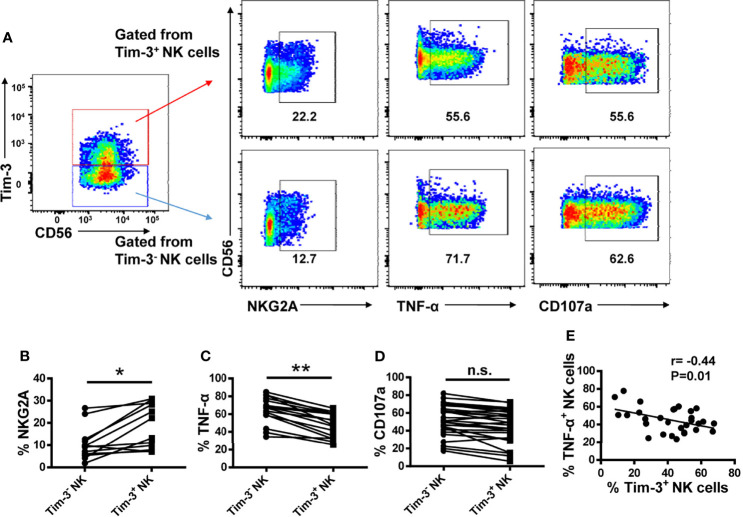
Tim-3^+^ NK cells display weaker activities than Tim-3^−^ NK cells in patients with T2DM. **(A)** Representative flow cytometry plots of NKG2A, TNF-α and CD107a and expression on Tim-3^+^ and Tim-3^−^ NK cells. **(B–D)** Proportions of NKG2A^+^ **(B)**, TNF-α^+^ **(C)**, and CD107a^+^ **(D)** Tim-3^+^ and Tim-3^−^ NK cells. **(E)** Analysis of the correlation between TNF-α levels and the percentage of Tim-3^+^ NK cells in patients with T2DM (Spearman’s correlation test). *P < 0.05, **P < 0.01, n.s., not significant.

To determine whether Tim-3 overexpression impairs NK cell function, we compared the expression of NKG2A, TNF-α, and CD107a in Tim-3^+^ and Tim-3^-^ NK cells from T2DM patients. We found that NKG2A expression was higher in Tim-3^+^, compared to Tim-3^-^, NK cells (**Figures 6A, B**). In addition, Tim-3^+^ NK cells produced significantly less TNF-α in response to PMA and ionomycin stimulation (**Figures 6A, C**) and showed also decreased CD107a production, although this change did not achieve statistical significance (**Figures 6A, D**). Furthermore, we found that Tim-3^+^ CD4^+^ T cells from T2DM patients have a much weaker capacity to produce TNF-α and IFN-γ after PMA/ionomycin induction (**Supplementary Figure S5A**). Importantly, a negative correlation was established between the percentage of TNF-α^+^ NK cells and that of Tim-3^+^ NK cells in patients with T2DM (r=–0.44, P=0.01; **Figure 6E**). These data indicate that Tim-3 overexpression is correlated with NK cell exhaustion in patients with long-term T2DM.

### Galectin-9/Tim-3 Pathway Blockade Restores NK Cell Function

It has been reported that Tim-3 exerts inhibitory function in T cells by interacting with Galectin-9, while the reports on CD66a/Tim-3 interaction remain conflicting (16, 28). No significant differences in Galectin-9 and CD66a/c/d/e expression were detected among monocytes, CD4^+^ T cells, CD8^+^ T cells, NK cells, NKT cells and dendritic cells from T2DM and control study participants (**Supplementary Figures S6A–D**). Consistent with previous studies (24, 29), we observed elevated serum Galectin-9 levels in patients with T2DM (**Supplementary Figure S6E**), suggesting the soluble Galectin-9 may negatively regulate the immune response *via* Galectin-9/Tim-3 pathway.

Then, we evaluated the influence of Galectin-9/Tim-3 or CD66a/Tim-3 interaction on NK cell functions using NK92 cell line. Mild expression of Tim-3 on NK92 cells were confirmed using flow cytometry. K562 cells, the commonly used NK target cells, rarely express Galectin-9 or CD66a/c/d/e. We constructed K562-Galectin-9 and K562-CD66a cells by overexpressing the respective receptors and expression of Galectin-9 and CD66a was confirmed with flow cytometry (**Supplementary Figure S7A**). K562, K562-Galectin-9 and K562-CD66a cells were subjected to NK92-mediated 4 hour cytotoxicity assay or 24h coculture assay. Overexpression of Galectin-9 on K562 cells significantly inhibited NK mediated cytotoxicity in 4h cytotoxicity assay as well as IFN-γ production in 24h coculture assay, while overexpression of CD66a has no impact (**Supplementary Figures S7B, C**). Overall, our results suggested that Galectin-9/Tim-3 interaction significantly inhibited NK cell function while CD66a/Tim-3 interaction had rare impact on NK cell function.

**Figure 7:**
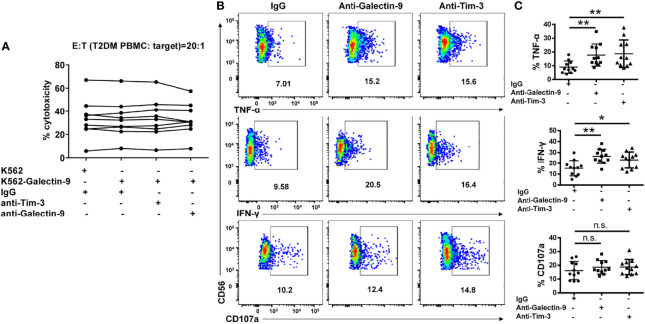
Galectin-9/Tim-3 pathway blockade restores cytokine production in NK cells from T2DM patients but do not affect T2DM NK mediated cell cytotoxicity. **(A)** PBMCs from T2DM patients were subjected to 4h cytotoxicity assay against K562 and K562-Galectin-9 cells in the presence or absence of Tim-3 and Galectin-9 antibodies. **(B)** PBMCs from T2DM patients were pretreated with Tim-3 or galectin-9 neutralizing antibodies and stimulated with IL12 and IL18 for evaluating cytokine secretion and granule release. Representative flow cytometry plots. **(C)** Quantification data. *P < 0.05, **P < 0.01, n.s., not significant.

We further assessed the influence of Galectin-9/Tim-3 blockade on T2DM NK cells function. We found that overexpression of Galectin-9 on K562 cells had no impact on T2DM NK cells mediated cytotoxicity and blocking Galectin-9/Tim-3 interaction with antibodies did not affect T2DM NK cells mediated cytotoxicity against K562-Galectin-9 (**Figure 7A**). In the IL-12 and IL-18 stimulating assay, it was observed that the production of both TNF-α and IFN-γ were upregulated upon Tim-3 and Galectin-9 blockade, while the production of CD107a did not achieve statistical significance (**Figures 7B, C**). Our results suggested that Tim-3 and Galectin-9 blockade recovered IFN-γ and TNF-α production of T2DM NK cells but did not affect T2DM NK cell-mediated cytotoxicity.

## Discussion

T2DM is associated with increased morbidity and mortality from several cancers and is also a risk factor for infectious diseases such as tuberculosis, melioidosis, and COVID-19 (30–32). Immune dysfunction is thought to be responsible for increased risk of infection and cancer incidence in people with T2DM. Previous studies have shown that T2DM impairs the immune responses of neutrophils, macrophages, and T cell (33). Hyperglycemia decreases the production of type I IFN, while low intracellular GSH concentrations reduce IL-12 and IFN-γ production, leaving patients vulnerable to multiple pathogens (34, 35).

NK cells play an important role in immune surveillance against infections and tumor cells. Since our understanding of the mechanisms underlying immune dysfunction in diabetes is still fragmentary, in this study we investigated the representation and functional status of NK cells in patients with T2DM. We found that both the frequency and absolute number of NK cells were significantly reduced in the peripheral blood of T2DM patients, and these changes were accompanied by overexpression of the inhibitory receptor Tim-3 and underexpression of the activating receptor NKG2D in those cells. Both TNF-α secretion and degranulation function (evidenced by CD107a expression) were dampened in patients’ NK cells. We further found that the expression of Tim-3 on NK cells positively correlated with patients HbA1c and FBG levels. Moreover, Tim-3 expression on NK cells negatively correlated with the percentage and absolute number of circulating total NK cells, and rendered them prone to apoptosis. In addition, Tim-3 expression on NK cells correlated negatively with their ability to secrete TNF-α and the function of NK cells could be restored by blocking Galectin-9/Tim-3 pathway.

Several studies reported no difference in the frequency of circulating NK cells between T2DM patients and non-diabetic controls (11, 36–40). Contrary to our findings, Lv et al. reported a significant increase in the proportion of NK cells in T2DM patients (41). It is possible that differences in inclusion criteria or testing methods may have led to these disparate conclusions. NK cells comprise a heterogeneous population, and changes in their activation status were reported at different stages of human type I diabetes (42).

Our study also provides a plausible explanation for the observed decrease in NK cell numbers in patients with T2DM, as Tim-3^+^ NK cells exhibited a greater tendency toward apoptosis than Tim-3^−^ NK cells, and their numbers correlated inversely with the abundance of total circulating NK cells. This is consistent with findings from Zheng et al. who found a negative correlation between Tim-3 expression and frequency of tumor-infiltrating NK cells (43). Indeed, Tim-3 has been reported to mediate T cell and NK cell apoptosis and dysfunction in various diseases (15, 44). In patients with atherosclerosis, increased expression of CD160 on NK cells stimulates the production of TNF-α, which leads to NK cell apoptosis and a subsequent decrease in the number of circulating NK cells (45).

We further found that T2DM-associated Tim-3 overexpression occurred in parallel with decreased NKG2D expression in both CD56^dim^ and CD56^bright^ NK cell subpopulations. Our findings are thus consistent with those of Berrou et al. who reported reduced expression of NKG2D and defective degranulation capacity in NK cells from T2DM patients (11). NKG2D expression is also reduced in NK cells from children with type 1 diabetes (T1D) and is linked to defective NKG2D-mediated activation of the phosphoinositide 3-kinase-AKT pathway (46). This suggests that NK cell dysfunction contributes to the pathogenesis of both T2DM and T1D. It was also reported that the activity of NK cells in T2DM patients is lower than in individuals with either prediabetes or normal glucose tolerance (40).

Despite the above evidence, the mechanisms underlying NK cell dysfunction in T2DM are not fully understood. As an inhibitory molecule, Tim-3 has been reported to promote immune tolerance through downregulation of Th1-dependent immune responses, and Tim-3 pathway blockade was shown to accelerate diabetes in non-obese diabetic mice (47) I contrast, and attesting to the obvious complexity of Tim-3 interactions in different immune cells, Tim-3 was shown to promote macrophage activation through the NF-κB/TNF-α pathway to exacerbate foot cell injury in diabetic nephropathy (48). In the present study, we demonstrated that the expression of Tim-3 on NK cells positively correlated with both HbA1c and FBG levels, two major markers of glycemic control. In addition, Tim-3 expression was associated with increased NKG2A levels in T2DM NK cells. Elevated expression of NKG2A, an immune checkpoint protein, was shown to induce NK cell exhaustion (49). Combined with the above results, our findings strongly connect Tim-3 expression with functional dysfunction of NK cells in T2DM.

The mechanisms regulating Tim-3 expression on NK cells remain incompletely understood. We found that high glucose treatment does not affect Tim-3 expression on NK cells *in vitro*, suggesting that hyperglycemia upregulating Tim-3 on NK cells indirectly, which needs to be further investigated. Galectin-9 is produced by diverse cell types, including tumor cells, endothelial cells and lymphocytes, and interacts with Tim-3 to negatively regulate cellular immune responses (50). We failed to detect significant differences in Galectin-9 expression in T cells, monocytes, dendritic cells and NKT cells from T2DM patients and healthy volunteers, but we found higher serum Galectin-9 levels in T2DM patients. The exact mechanism of secretion for Galectin-9 is poorly understood and it is not certain which cells are responsible for the elevated serum soluble Galectin-9 (50), while cells other than immune cells might contribute to it (51, 52).

CD66a has been reported to be a ligand for Tim-3 and endows Tim-3 with inhibitory function to suppress T cell function and downregulate anti-tumor immunity (16). But the reports remain conflicting and need to be further investigated. It was previously reported that the level of CD66a in serum was significantly decreased in T2DM patients (53). In our study, we found there were no significant differences in CD66a/c/d/e expression on monocytes, T cells, NK cells, NKT cells and dendritic cells from T2DM and healthy donors. Meanwhile, we explored the influence of CD66a/Tim-3 interaction on NK cell function and found that overexpression of CD66a on target cells did not affect NK92 cell mediated cytotoxicity or IFN-γ production, suggesting CD66a/Tim-3 has rare impact on NK cell function.

Our results showed that Tim-3 and Galectin-9 blockade recovered IFN-γ and TNF-α production but did not affect CD107a expression, which is consistent with the results that overexpression of Galectin-9 on K562 cells does not affect T2DM NK cell-mediated cytotoxicity.

In conclusion, this study comprehensively assessed the expression of key immunomodulatory receptors and cytokines on NK cells and provided new insights into the phenotypic alterations that drive impaired NK cell functions in patients with T2DM. Our data suggest that elevated Tim-3 expression on NK cells leads to apoptosis-mediated reduction in NK cell numbers and induces NK cell dysfunction, thereby increasing the risk of cancer and infectious diseases in T2DM.

## Data Availability Statement

The original contributions presented in the study are included in the article/**Supplementary Material**. Further inquiries can be directed to the corresponding author.

## Ethics Statement

The studies involving human participants were reviewed and approved by The First Affiliated Hospital of Anhui Medical University The committee on Medical Ethics. The patients/participants provided their written informed consent to participate in this study. Written informed consent was obtained from the individual(s) for the publication of any potentially identifiable images or data included in this article.

## Author Contributions

HW and LT designed and wrote the manuscript. HW and SL performed the experiments and analyses. KC and YX were involved in the collection of clinical samples. LT designed and supervised the experiments and wrote the manuscript. All authors contributed to the article and approved the submitted version.

## Funding

This work was supported by the National Natural Science Foundation of China (Grant No. 31800737).

## Conflict of Interest

The authors declare that the research was conducted in the absence of any commercial or financial relationships that could be construed as a potential conflict of interest.

## Publisher’s Note

All claims expressed in this article are solely those of the authors and do not necessarily represent those of their affiliated organizations, or those of the publisher, the editors and the reviewers. Any product that may be evaluated in this article, or claim that may be made by its manufacturer, is not guaranteed or endorsed by the publisher.
