# Supplementary material for: Tim-3 Expression Causes NK Cell Dysfunction in Type 2 Diabetes Patients
Source: Front Immunol. 2022 Apr 5;13:852436. doi: 10.3389/fimmu.2022.852436 (PMC9018664; doi:10.3389/fimmu.2022.852436)
Supplement: Supplementary file 1 [file DataSheet_1.docx]

**Supplementary Table**

**Table S1**. Clinical characteristics of enrolled subjects

**Clinical characteristics** **T2DM**  **Healthy controls**

Case 90 62

Sex (male) 51 (56.7%) 33 (53.2%)

Age, year 59.24 ± 9.79 56.61 ± 7.83

HbA1c, % 9.79 ± 1.79 5.26 ± 0.22

FBG, mmol/L 11.88 ± 3.97 5.30 ± 0.32

TC, mmol/L 4.47 ± 1.17 4.94 ± 0.93

TG, mmol/L 1.96 ± 1.16 1.39 ± 0.77

HDL, mmol/L 1.03 ± 0.29 1.43 ± 0.38

LDL, mmol/L 2.71 ± 0.97 2.80 ± 0.80

Creatinine, µmol/L 66.51 ± 25.72 65.13 ± 14.26

UA, µmol/L 299.0 ± 86.86 329.3 ± 79.71

Data were expressed as mean ± standard deviation (SD). Abbreviations: FBG: fasting blood glucose; TC: serum total cholesterol; TG: serum triglyceride; HDL: highdensity lipoprotein; LDL: low-density lipoprotein; UA: uric acid

**Supplementary Figures**

**Figure S1 JPG**


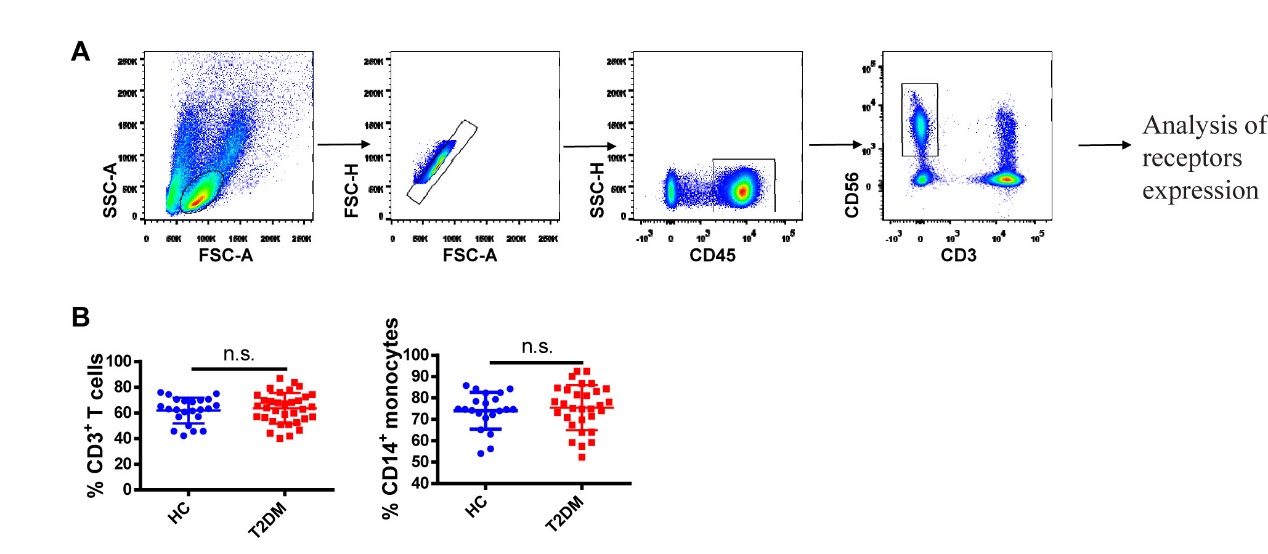


**Figure S1:** (**A**) Sequential strategy for gating NK cells from PBMCs via flow cytometry. (**B**) Frequency of CD3^+^ T cells and CD14^+^ monocytes from patients with T2DM and healthy controls (HC). Each dot represents a different individual and results are presented as the mean ± SEM; n.s., not significant.

**Figure S2 JPG**


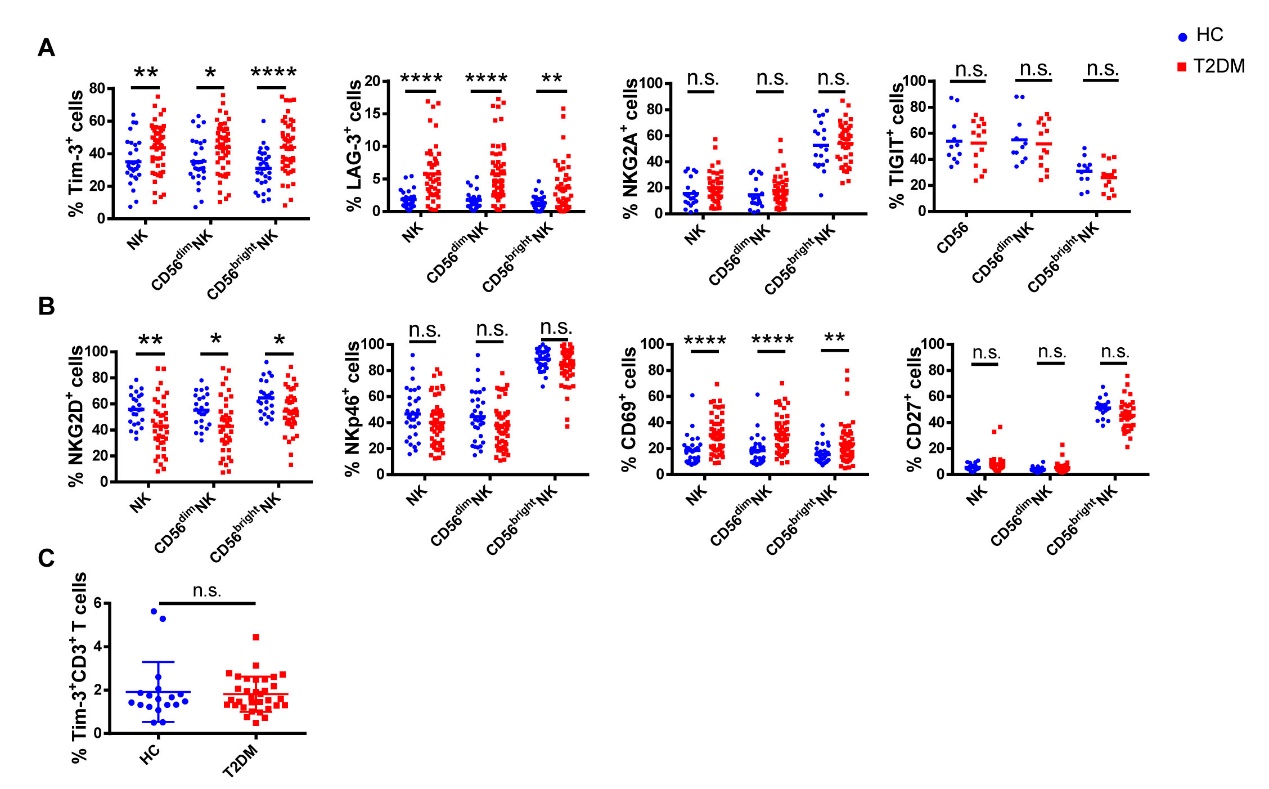


**Figure S2: NK cells from T2DM patients overexpress Tim-3 and underexpress NKG2D.** (**A**) Quantification of the expression of inhibitory receptors (Tim-3, LAG-3, NKG2A, and TIGIT) on NK cells from patients with T2DM and healthy controls (HC). (**B**) Quantification of the expression of activating receptors (NKG2D, NKp46, CD69, and CD27) on NK cells from patients with T2DM and HC. (**C**) Expression of Tim-3 on CD3^+^ T cells from patients with T2DM and HC. Data are representative of more than three independent experiments and results are presented as the mean ± SEM; *p<0.05, **p<0.01, ***p<0.001, ****P<0.0001, n.s., not significant.

**Figure S3 JPG**


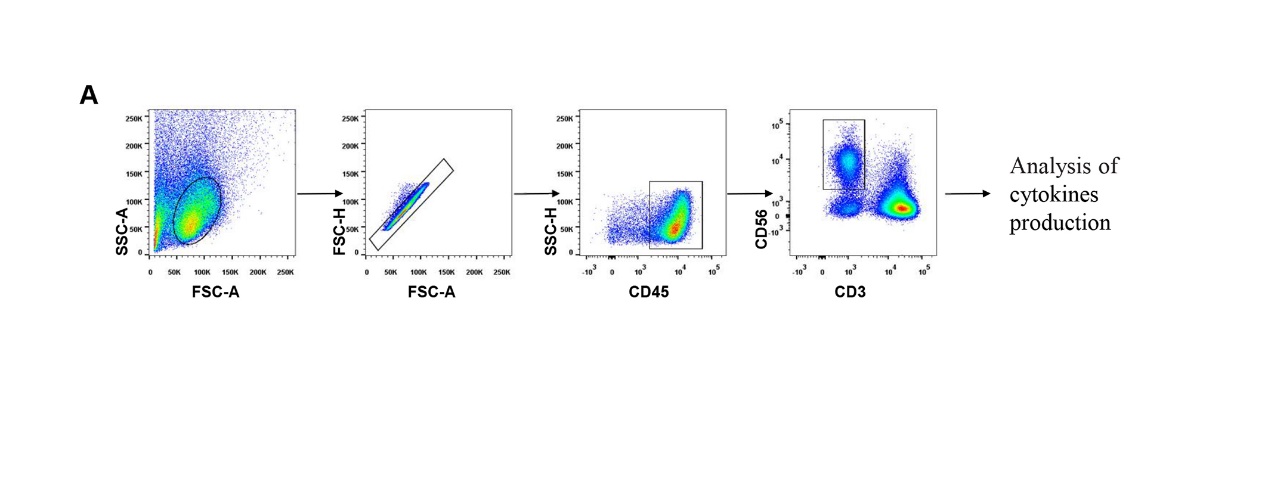


**Figure S3:** (**A**) Sequential strategy for gating NK cells from PBMCs for cytokine production analysis via flow cytometry.

**Figure S4 JPG**


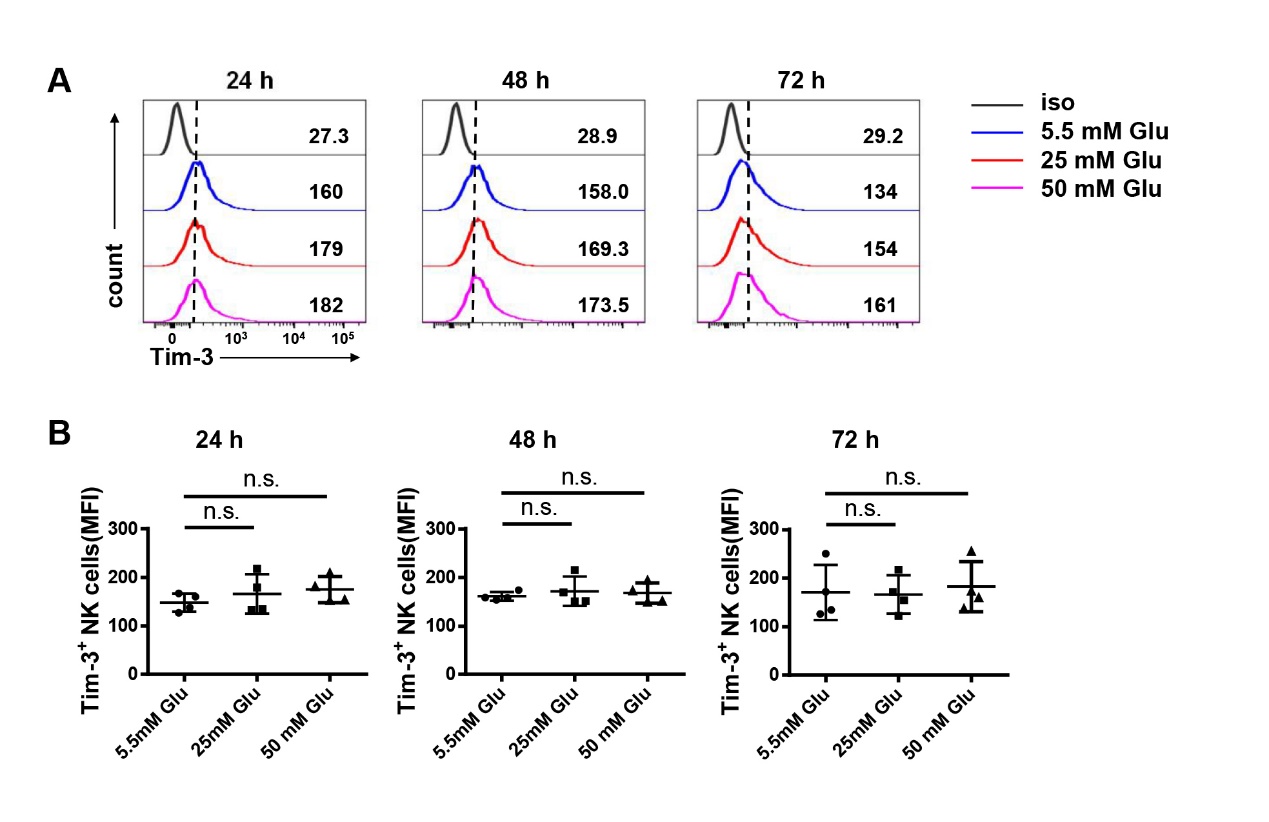


**Figure S4:** High glucose treatment does not affect Tim-3 expression on NK cells. PBMCs from healthy donors were cultured in RPMI-1640 medium containing 5.5 mM, 25 mM and 50 mM glucose. Expression of Tim-3 on NK cells after incubation 24h, 48h and 72h (**A**, **B**). n.s., not significant.

**Figure S5 JPG**


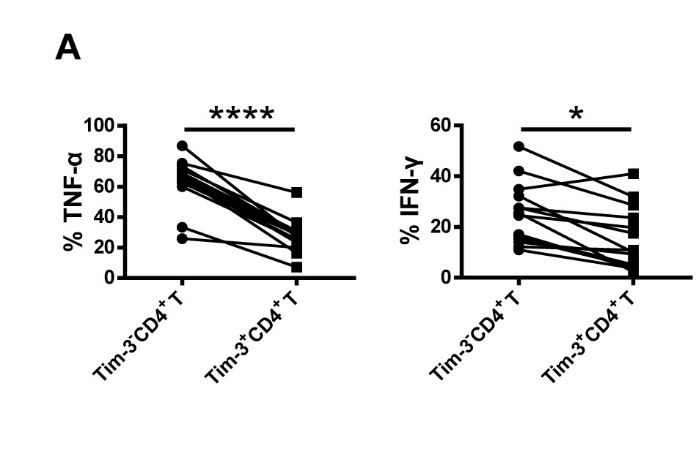


**Figure S5:** (**A**) Proportions of TNF-α^+^ and IFN-γ^+^ cells in Tim-3^+^ CD4^+^ T cells and Tim-3^−^ CD4^+^ T cells. Each symbol represents data from an individual patient. *p<0.05, **p<0.01, ***p<0.001, ****p<0.0001, n.s., not significant.

**Figure S6 JPG**


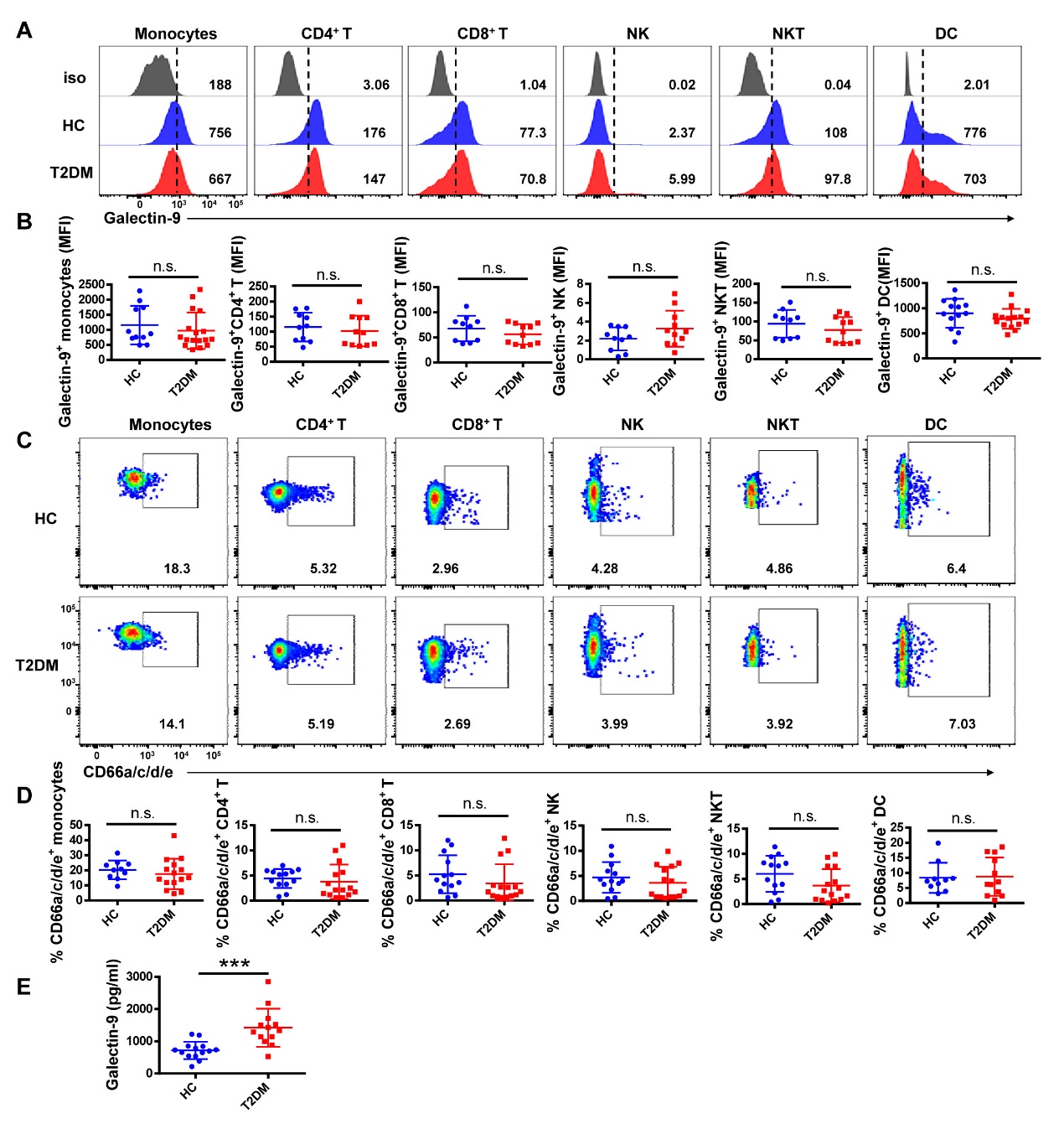


**Figure S6:** Representative histograms showing the expression of the (**A**) galectin-9 and (**C**) CD66a/c/d/e on CD14^+^ monocytes, CD4^+^ T, CD8^+^ T, NK, NKT cells and lin^-^ HLA-DR^+^ dendritic cells (DCs) from patients with T2DM and healthy controls (HC). Comparison of the MFI of (**B**) galectin-9 and quantification of (**D**) CD66 a/c/d/e in on monocytes, CD4^+^ T, CD8^+^ T, NK, NKT and DCs from T2DM patients and HC. (**E**) Dot plots of galectin-9 concentrations in serum samples from health individuals and patients with T2DM. ***P<0.001, n.s., not significant.

**Figure S7 JPG**


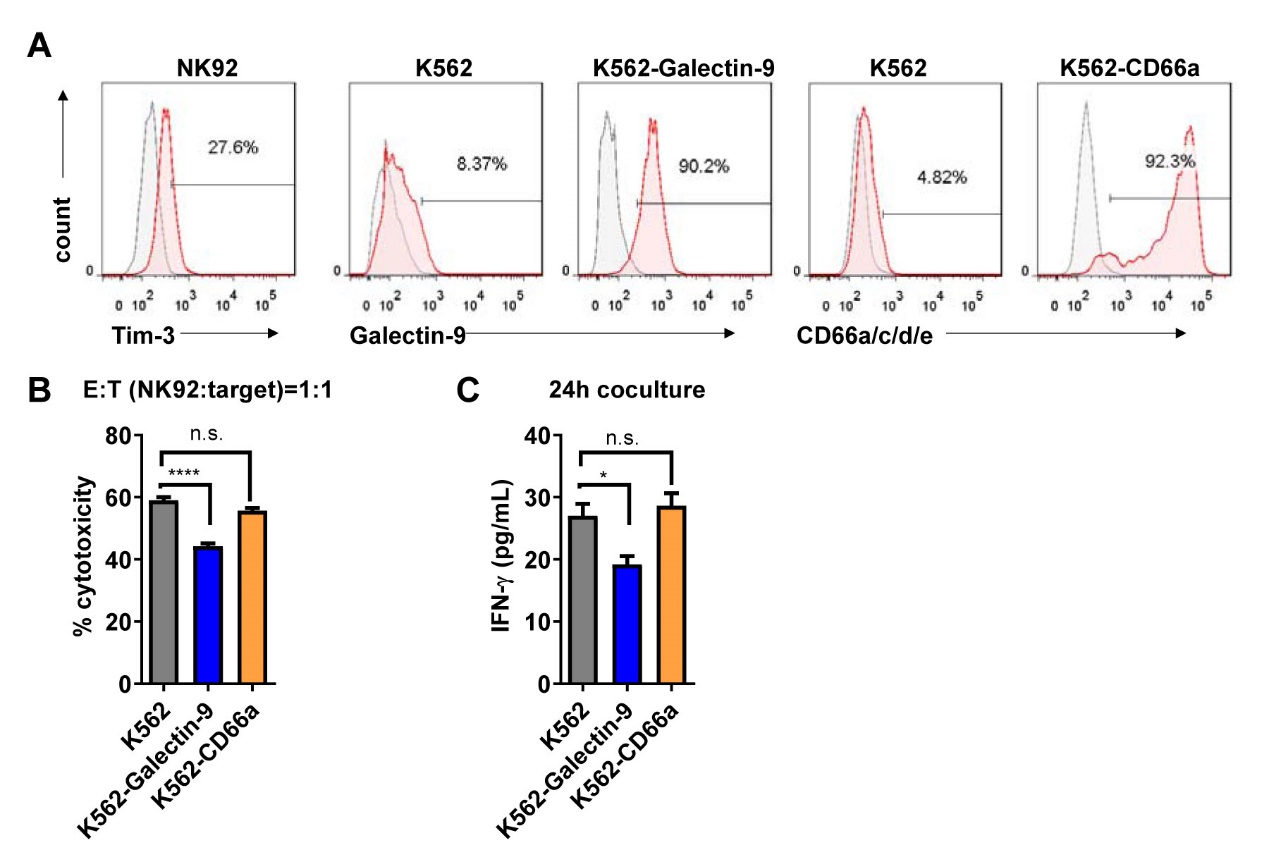


**Figure S7: Galectin-9/Tim-3 but not CD66a/Tim-3 interaction inhibits NK92 mediated cell cytotoxicity and IFN-γ production.** (**A**)Expression of Tim-3 on NK92 cells, Galectin-9 on K562 and K562-Galectin-9 cells, CD66a/c/d/e on K562 and K562-CD66a cells are assessed by flow cytometry. (**B**) K562, K562-Galectin-9 and K562-CD66a cells were subjected to NK92 cell mediated cytotoxicity assay at the effector: target (E:T) ration of 1:1. (**C**) K562, K562-Galectin-9 and K562-CD66a cells were cocultured with NK92 cells for 24h and IFN-γ in culturing supernate were analyzed by ELISA. Quantification data. *P<0.05, ****P<0.0001, n.s., not significant.
